# The interactions of novel mononuclear platinum-based complexes with DNA

**DOI:** 10.1186/s12885-018-5194-8

**Published:** 2018-12-22

**Authors:** Ben W. Johnson, Mark W. Burgess, Vincent Murray, Janice R. Aldrich-Wright, Mark D. Temple

**Affiliations:** 1 School of Science and Health, Western Sydney University, Campbelltown, NSW 2560 Australia; 2 School of Biotechnology and Biomolecular Sciences, University of New South Wales, Sydney, Australia

**Keywords:** Anticancer drug, Cisplatin, DNA adducts, Interstrand cross-linking, Sequence specificity, Linear amplification reaction, Telomeric repeat

## Abstract

**Background:**

Cisplatin has been widely used for the treatment of cancer and its antitumour activity is attributed to its capacity to form DNA adducts, predominantly at guanine residues, which impede cellular processes such as DNA replication and transcription. However, there are associated toxicity and drug resistance issues which plague its use. This has prompted the development and screening of a range of chemotherapeutic drug analogues towards improved efficacy. The biological properties of three novel platinum-based compounds consisting of varying *cis*-configured ligand groups, as well as a commercially supplied compound, were characterised in this study to determine their potential as anticancer agents.

**Methods:**

The linear amplification reaction was employed, in conjunction with capillary electrophoresis, to quantify the sequence specificity of DNA adducts induced by these compounds using a DNA template containing telomeric repeat sequences. Additionally, the DNA interstrand cross-linking and unwinding efficiency of these compounds were assessed through the application of denaturing and native agarose gel electrophoresis techniques, respectively. Their cytotoxicity was determined in HeLa cells using a colorimetric cell viability assay.

**Results:**

All three novel platinum-based compounds were found to induce DNA adduct formation at the tandem telomeric repeat sequences. The sequence specificity profile at these sites was characterised and these were distinct from that of cisplatin. Two of these compounds with the enantiomeric 1,2-diaminocyclopentane ligand (*SS* and *RR*-DACP) were found to induce a greater degree of DNA unwinding than cisplatin, but exhibited marginally lower DNA cross-linking efficiencies. Furthermore, the *RR*-isomer was more cytotoxic in HeLa cells than cisplatin.

**Conclusions:**

The biological characteristics of these compounds were assessed relative to cisplatin, and a variation in the sequence specificity and a greater capacity to induce DNA unwinding was observed. These compounds warrant further investigations towards developing more efficient chemotherapeutic drugs.

## Background

**Fig. 1:**
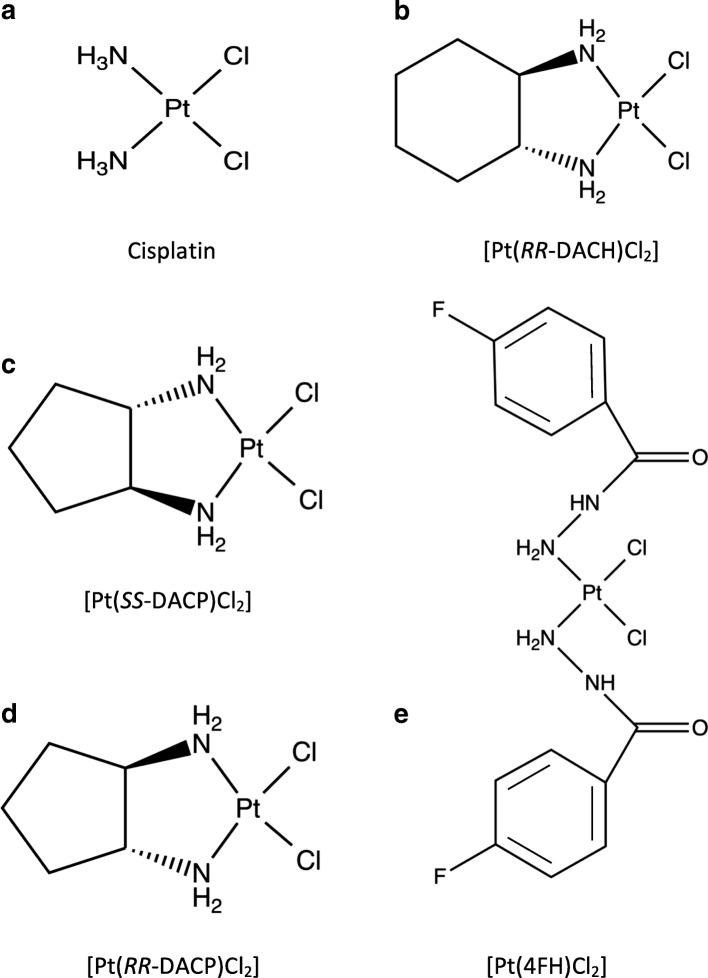
The chemical structures of cisplatin, *RR*-DACH and the three novel platinum-based compounds used in this study. All compounds used in this study consist of a single platinum atom and two chloride atoms arranged in the *cis* configuration. Note that (**a**) cisplatin is a square planar compound containing two ammine groups attached to the central platinum atom. *RR*-DACH, contains an *RR*-configured cyclohexane ligand attached to two ammine groups. The novel compounds (**c**) [Pt(*SS*-DACP)Cl_2_] and (**d**) [Pt(*RR*-DACP)Cl_2_] consist of isomeric configured cyclopentane ligands. The novel compound (**e**) [Pt(4FH)Cl_2_] contains symmetrical fluorobenzoic hydrazide groups attached to ammine groups on either side of the platinum atom

*cis-*Diamminedichloroplatinum(II) (cisplatin) is a square planar compound consisting of a central platinum atom (Fig. 1a) that is widely used in cancer chemotherapy. Its key biological target is DNA [1] where it forms either a monofunctional adduct through covalent interactions between a purine base and the monoaquated cisplatin species or a bifunctional adduct between two purine bases and the diaquated species [2, 3]. Intrastrand DNA adducts between adjacent G residues account for 60% of cisplatin’s covalent interaction [4] and these contribute significantly to its biological and antitumour activity, in addition to DNA adducts formed at AG, GA and GC sequences [5, 6]. Cisplatin also forms interstrand adducts between two offset guanine bases (on opposing strands of the DNA helix), which account for just 2% of adducts [4] but these are the most toxic and are thought to hinder DNA replication and transcription, leading to cell cycle arrest and apoptosis [6–8].

Located at the ends of chromosomes are G-rich telomere sequences consisting of tandem repeats of 5′-TTAGGG_(n)_-3′ [9]. These undergo shortening during progressive rounds of normal cell replication that limits cell lifespan. Expression of the telomerase enzyme in malignant cells catalyses the extension of these DNA sequences, contributing to cellular immortality associated with carcinogenesis [10]. DNA templates consisting of eukaryotic telomere sequences are preferential targets for DNA damage induced by cisplatin and other novel platinum-based compounds [11–13]. Furthermore, cisplatin-induced DNA adducts at telomeres have been correlated to a regression in tumour size in patients with advanced head and neck cancer [14]. Hence, the ability of novel platinum-based anticancer drugs to form DNA adducts at telomere sequences may be a desirable property to assess in relation to their potential use in cancer therapy.

Cisplatin-induced DNA adducts have the ability to bend and locally unwind the DNA helix to cause unstacking of the guanines [15] and to kink DNA by 53° [16]. These adducts are recognised by enzymes including those involved with DNA repair [17], such as the nucleotide excision repair proteins [18, 19]. Alternatively, DNA lesions may be shielded by HMG box 1 proteins [20, 21] that protect them from repair and this may be a key factor contributing to their antitumour efficiency.

Cisplatin is used for the treatment of various cancers, however, there are a range of associated cytotoxicity and resistance issues that may be overcome by the design and synthesise of novel platinum-based analogues. Since its discovery, over 3000 platinum-based compounds have been synthesised but only 35 of these have proven advantages relative to cisplatin for use in clinical practice [22]. One of the most successful of these is 1*R*,2*R*-diaminocyclohexaneoxalatoplatinum(II) (oxaliplatin). Oxaliplatin exhibits an enhanced range of activity and a lower cross-resistance to that of cisplatin and the presence of the 1*R*,2*R*-diaminocyclohexane (DACH) ligand may be responsible for this [23]. As a case in point, oxaliplatin is highly effective in the treatment of colon cancer whilst cisplatin has minimal effect [22]. Additionally, use of oxaliplatin is associated with lower toxicity and higher patient survival rates than cisplatin in the treatment of advanced, unresectable gastric cancer [24]. Since the emergence of oxaliplatin, a range of novel platinum-based compounds containing a 1*S*,2*S*-diaminocyclohexane ligand, such as 56MESS, have been shown to be more cytotoxic than cisplatin in a range of cisplatin-sensitive and cisplatin-resistant cell lines [25]. In this study, the biological properties of three novel platinum-based compounds consisting of varying *cis*-configured ligand groups as well as a commercially supplied compound were characterised to determine their potential as anticancer agents.

## Methods

**Fig. 2:**
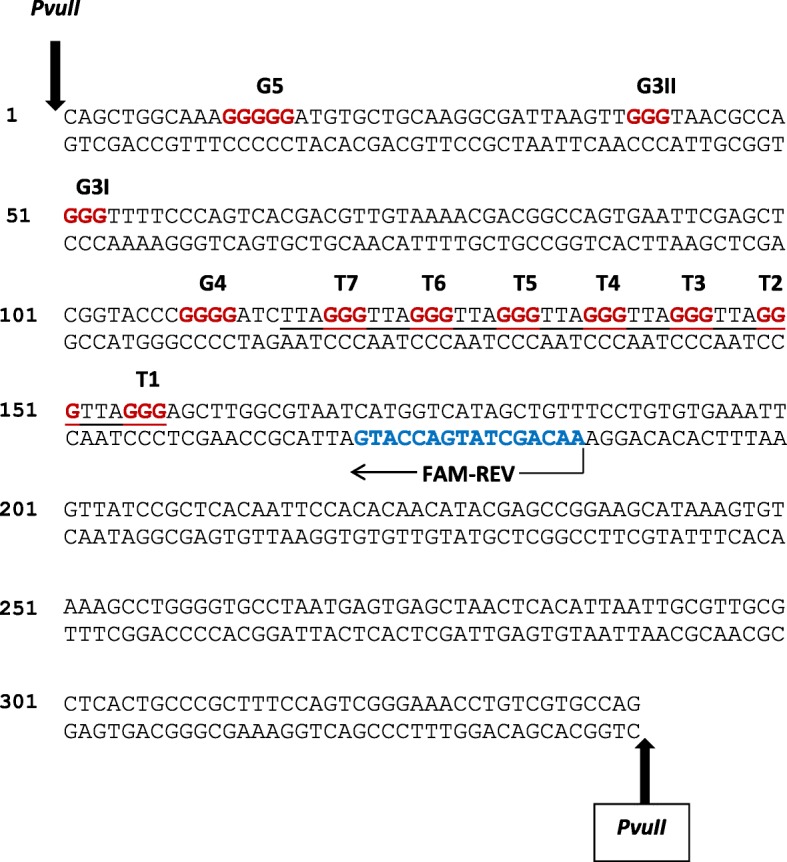
Double-stranded sequence of the *Pvu*II-cleaved pUC19/T7 DNA template. The upper strand of the sequence is written in the 5′ to 3′ direction. Sites of *Pvu*II restriction enzyme cleavage are indicated by the bold vertical arrows and the annealing site of the FAM-REV primer is shown by a horizontal arrow, with the primer sequence highlighted in blue. The seven telomeric repeats sequences (T1 to T7) are underlined, with the guanines highlighted in red. Other sites of three or more consecutive guanines (G3I, G3II, G4 and G5) are highlighted in red

In this investigation, we report the biological properties of four platinum-based compounds [Pt(*RR*-DACH)Cl_2_], [Pt(*RR*-DACP)Cl_2_], [Pt(*SS*-DACP)Cl_2_] and [Pt(4FH)Cl_2_], as shown in Fig. 1. *RR*-DACH is structurally related to oxaliplatin whereas the isomers *RR*-DACP and *SS*-DACP contain one less carbon in the cyclic ring. The DNA sequence used to characterise the sites of damage contained several telomeric repeat sites, as shown in Fig. 2. This was achieved using a modified linear amplification reaction (LAR) [26] to detect and quantify DNA damage (bulky DNA adducts) at single nucleotide resolution. This technique involves *Taq* DNA polymerase extending from a fluorescently-labelled oligonucleotide primer until its activity is terminated by a DNA adduct [27, 28]. Fluorescently-labelled DNA fragments were analysed using a capillary sequencer [12, 29]. This method is more efficient and accurate than conventional gel electrophoresis for the quantification of data [30, 31]. Additionally, this research characterises the DNA interstrand cross-linking and DNA unwinding efficiencies of the platinum-based compounds, using a purified pUC19 DNA template, through the application of denaturing and native agarose gel electrophoresis techniques, respectively.

### Chemicals and starting materials

Cisplatin and the ligand, *RR*-DACH, were purchased from Sigma-Aldrich. The novel platinum-based compounds, [Pt(*RR*-DACP)Cl_2_], [Pt(*SS*-DACP)Cl_2_] and [Pt(4FH)Cl_2_], were synthesised and provided as a kind gift by Janice Aldrich Wright of the Western Sydney University, Sydney, Australia. All compounds were dissolved in dimethylformamide (DMF) to give working stock solutions of 5 mM prior to use.

DH5α *E. coli* cells transfected with pUC19, with an insert of seven telomeric repeat sequences (pUC19/T7) between the *Bam*HI and *Hin*dIII restriction enzyme sites [32], were utilised for this study. Purified pUC19/T7 plasmid derived DNA was used for all experiments involving DNA-drug interactions. The 5’ FAM-labelled reverse sequencing primer (FAM-REV) 5’-AACAGCTATGACCATG-3’ [32] used in the LAR procedure was purchased HPLC purified from Invitrogen.

### DNA preparation

The pUC19/T7 DNA was extracted and purified from the DH5α *E. coli* cells, using a Qiagen Plasmid Maxi purification kit. The purified plasmid pUC19/T7 DNA was linearised with a *Pvu*II restriction enzyme prior to the DNA damage reactions for the LAR and interstrand cross-linking assays. The native undigested pUC19/T7 DNA was used in the DNA unwinding assay.

### DNA damage reactions

DNA damage reactions were carried out by treating 500 or 800 ng of the native or *Pvu*II-cleaved pUC19/T7 DNA, respectively, with increasing concentrations of each compound. The samples were prepared in a final reaction volume of 40 μL, consisting of 2 mM *N*-2-hydroxyethylpiperazine-*N′*-2-ethane sulfonic acid (HEPES) (pH 7.8), 10 mM NaCl and 10 μM EDTA, incubated at 37 °C for 18 h in the dark. A DMF solvent control was prepared and incubated under the same conditions as the drug-treated samples by substituting the drug with 5% (*v*/v) DMF. Following incubation, an ethanol precipitation was performed on all samples and the DNA pellets were re-dissolved in 20 μL of 10 mM Tris-HCl (pH 8.8), 0.1 mM Na_2_EDTA. The *Pvu*II-cleaved pUC19/T7 DNA samples were used in the LAR and interstrand cross-linking assays as previously described [33]. All assays were reproduced in triplicate to give consistent results, representative examples are shown.

### DNA unwinding assay

Native agarose gels were prepared at 1% (*w*/*v*) and submerged in TAE running buffer, comprising of 40 mM Tris-acetate and 1 mM EDTA (pH 8). The gel loading buffer consisted of 30% (*v*/v) glycerol and 0.25% (w/v) bromophenol blue in DNase-free water. The loading buffer was combined with ~ 500 ng of drug-treated native (un-cleaved) pUC19 DNA sample in a 1:5 ratio, made up to a final volume of 6.25 μL. The 1% (w/v) agarose gel were cast and run on a Bio-Rad Wide Mini-Sub Cell GT System with 6.25 μL drug-treated plasmid DNA samples or untreated plasmid DNA sample (DMF solvent control) and 5 μL of an Axygen Biosciences ready-to-use 1 kb DNA molecular weight ladder. Gel electrophoresis was carried out at 5.5 V/cm for 3 h, followed by staining in 1 x GelRed stain (diluted in 1 x TAE buffer, pH 8) for at least 30 min. The gel was visualised under UV light with a BioRad Gel Doc 2000 imager and compound-induced apparent molecular weight changes of the plasmid DNA were analysed against the 1 kb ladder standard, using BioRad Quantity One gel imaging software.

### Preparation of HeLa cells and cytotoxicity assay

HeLa cells were cultured as subconfluent monolayers in 75 cm^2^ culture flasks and maintained at 37 °C in 5% (*v*/v) CO_2_. The cells were subcultured in Dulbecco’s modified eagle medium (DMEM), supplemented with 10% (v/v) FBS, 4.5 g/L *D*-Glucose, *L*-Glutamine, 110 mg/L Sodium Pyruvate, 200 U/mL Penicillin and 200 μg/mL Streptomycin. The HeLa cells were harvested after 3 days or when at 90% confluence. Following trypsinisation the cell pellet was washed with 10 mL of Dulbecco’s phosphate buffered saline (DPBS) and centrifugation at 500 x g for 5 min. The DPBS was decanted and the cell pellet was resuspended in 10 mL of fresh DMEM media. A 10 μL aliquot was combined with 10 μL of 0.4% (*w*/*v*) trypan blue solution for cell counting, cells were diluted with DMEM media to yield a final concentration of 100,000 cells/mL. 100 μL aliquots were pipetted into the wells of a flat-bottomed 96-well microtitre plate and incubated at 37 °C with 5% (*v*/v) CO_2_, for 24 h. The DMEM culture media was replaced with 100 μL of fresh DMEM media containing the platinum-based compound at concentrations of 1, 3, 5, 10, 30, 50 and 100 μM. In the case of 4FH, the drug concentration was extended further to 150 and 200 μM. All drug treatments were carried out in triplicate and untreated cell controls (cells in DMEM without drug) were included for each experiment. 2% (*v*/v) DMF solvent controls were included in triplicate for each experiment. Triplicate blank controls (no cells) contained 100 μL DMEM media. The 96-well plate was incubated at 37 °C with 5% (v/v) CO_2_ for 24 h. Following drug treatment, 50 μL of a 5 mg/mL MTT-DPBS solution was added and cells incubated for 2 h at 37 °C with 5% (v/v) CO_2_, the media was removed from the wells by pipette and the resulting formazan crystals were solubilised in 100 μL of DMSO [34]. The plate was placed on a plate shaker for 30 min to ensure thorough solubilisation of the formazan crystals prior to measuring the optical density at 550 nm with a Thermo Scientific Multiskan EX microplate reader.

### Data analysis

The ssDNA products generated by the LAR procedure were analysed on an ABI 3730 capillary sequencer by fragment analysis, alongside reaction products obtained from dideoxy sequencing on the untreated *Pvu*II-cleaved pUC19/T7 DNA. For each nucleotide peak in the analysis trace the percentage of damage was determined using GeneMapper software and normalised to a maximal value of 1, relative to the highest percentage of damage.

The degree of drug-induced DNA cross-linking was determined by measuring the band intensity of the large fragment of the *Pvu*II-cleaved (linearised) pUC19, occurring as ssDNA and dsDNA forms in each lane of the denaturing agarose gels. The drug concentration that prevents 50% of the dsDNA from being denatured on the denaturing agarose gel was determined through the application of non-linear regression analysis using GraphPad Prism software. The frequency of interstrand cross-linking was calculated according to the formula; %ICL/Pt = XL/5408 x r_b_ [35]. Where %ICL/Pt refers to the percentage of interstrand crosslinks per platinum adduct and XL is the number of interstrand crosslinks per template molecule, XL = −ln *A* (A is the fraction of DNA molecules corresponding to the non-cross-linked ssDNA band) and 5408 corresponds to the number of nucleotide residues of the linearised DNA template. The r_b_ ratios were estimated from the molarity of platinum compound and nucleotides present in the solution at which 50% of the DNA was resistant to denaturing. This assumes that all of the drug has reacted with the DNA. This assumption is supported by the fact that whilst determining the sequence specificity there were still undamaged DNA sequences present and adding more platinum compound led to further damage [13].

The extent to which a compound could unwind the supercoiled conformation of pUC19 plasmid was observed through changes in the electrophoretic mobility of undigested plasmid conformations on the native agarose gels. The drug concentrations that caused open and supercoiled plasmid conformations to merge at a point of coalescence was determined by a linear regression analysis. The point of coalescence of the plasmid conformations was determined from the forecasted x-coordinates at which the two slopes intercepted each other. The experimentally determined coalescence points were used to calculate the associated DNA unwinding angle of the supercoiled conformation, induced by each compound, which were calculated using the formula, ϕ = 18σ / r_b_(c) [36]; whereby ϕ is the DNA unwinding angle, σ is the super-helical density of the supercoiled conformation and r_b_(c) is the drug to nucleotide ratio at the point of coalescence.

The degree of compound cytotoxicity (the IC_50_ value) was determined from analysis of the dose response curve. This involved the application of a non-linear regression analysis to identify the drug concentration that induced a 50% inhibition of cell viability using GraphPad Prism software.

## Results

In this investigation all compounds were evaluated for their cytotoxicity and further characterised for their DNA binding properties. It is of interest to characterise the DNA binding properties of all of these compounds regardless of their cytotoxicity since this may aid the identification of their structural attributes that are either beneficial or an impediment to their possible clinical potential. For instance, a compound may be highly reactive to purified DNA but may possess structural properties that hinder its capacity to reach DNA in a cellular environment. This may be useful knowledge to inform on the design of new compounds with both desirable DNA binding attributes and behaviour within a cellular environment. It is anticipated that a knowledge of these biological properties will enable the design and synthesis of a range of platinum-based drugs with desirable chemotherapeutic properties that surpass the clinical efficacy of cisplatin.

### Sequence specificity of the platinum-based compounds in the pUC19/T7 sequence

The pUC19/T7 sequence contains seven telomeric repeats consisting of GGGATT (T1-T7), a site of five consecutive guanine bases (G5), a site of four consecutive guanine bases (G4) and two sites of three consecutive guanine bases (G3I and G3II). These features and the annealing site of the FAM-REV primer are shown in Fig. 2. After treating the *Pvu*II-cleaved pUC19/T7 DNA samples with each compound, the LAR procedure was carried out with the FAM-REV primer, which yields a full length single-stranded DNA (ssDNA) product of 186 bp.

**Fig. 3:**
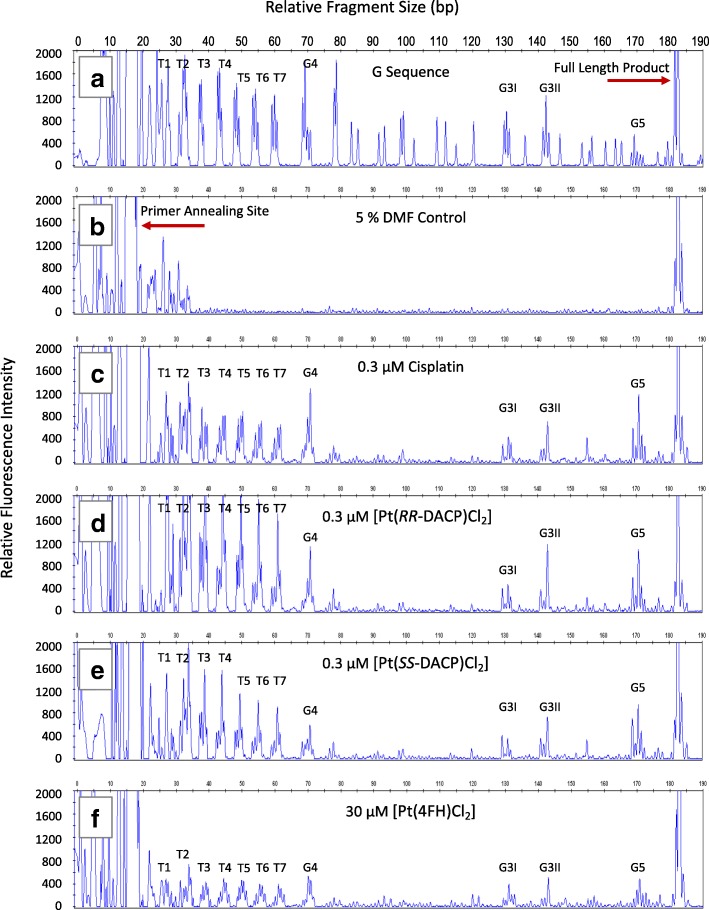
Electropherogram traces showing DNA damage induced by cisplatin and novel platinum-based compounds that was amplified by the LAR. **a** The G dideoxy sequencing lane used to determining the location of G-bases in the *Pvu*II-cleaved pUC19/T7 sequence. **b** The 5% (*v*/v) DMF treated DNA, a negative control. DNA damage induced by (**c**) 0.3 μM cisplatin, (**d**) 0.3 μM [Pt(*RR*-DACP)Cl_2_] (**e**) 0.3 μM [Pt(*SS*-DACP)Cl_2_] and (**f**) 30 μM [Pt(4FH)Cl_2_]. The relative fluorescence intensity is plotted on the y-axis (left) and the DNA fragment size (bp) is plotted on the x-axis (top). The primer annealing site and full length extension product of 186 bp are highlighted. Damage peaks corresponding to sites of telomeric repeats T1 to T7 and the G-rich sites (G3I, G3II, G4 and G5) are labelled accordingly

The LAR experimental conditions were optimised by treating the DNA template with an increasing concentration of each compound. Damage peaks in the electropherogram traces were identified by comparison to peaks in the untreated G dideoxy sequencing lane, as shown in Fig. 3a. Note that the LAR was not carried out for [Pt(*RR*-DACH)Cl_2_] as its sequence specificity has already been characterised [37]. For cisplatin, [Pt(*RR*-DACP)Cl_2_] and [Pt(*SS*-DACP)Cl_2_] an optimal concentration of 0.3 μM was found to yield an even distribution of damage across the entire length of the DNA template, whilst maintaining a strong signal corresponding to the full length extension product of 186 bp, as shown in Fig. 3c-e. However, a hundred-fold higher compound concentration of 30 μM was required for [Pt(4FH)Cl_2_] in order for it to produce observable DNA damage, as shown in Fig. 3f. Compound induced damage is predominantly observed at the telomeric repeat sequences, and other G-rich sites in the *Pvu*II-cleaved pUC19/T7 sequence, as shown in Fig. 3c-f. There was negligible damage in the DMF control sample as shown by the absence of background peaks in the DMF trace, shown in Fig. 3b. However, DNA damage could not be interpreted at the first telomeric repeat (T1) as a result of artefact peaks in close proximity to the primer annealing site, in some replicates there was also a lesser degree of interference at the T2 site.

**Fig. 4:**
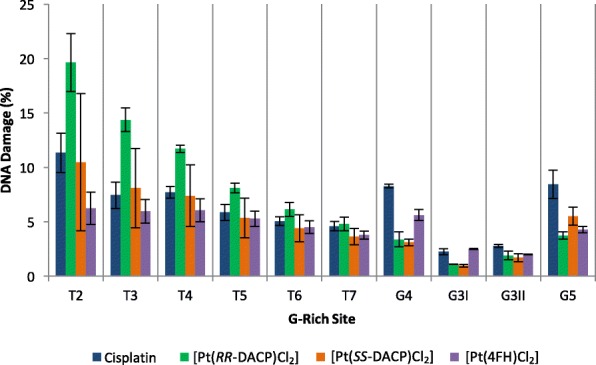
Histograms showing the overall percentage of DNA damage induced by each compound in the *Pvu*II-cleaved pUC19/T7 sequence. The percentage of overall DNA damage (y-axis) is shown for each G-rich site (x-axis). The overall DNA damage is the sum of damage at each individual base within the G-rich site. The error bars represent the SEM, determined from three separate experiments. The T1 site has been omitted as the DNA damage overlapped with artefact peaks present in the DMF control

Overall, the collective sum of T2-T7 telomeric repeats accounting for 32–65% of total damage within the pUC19/T7 sequence. All tested compounds, with the exception of [Pt(4FH)Cl_2_], exhibited the highest damage intensities at the T2-T4 telomeric sites (Fig. 4). [Pt(*RR*-DACP)Cl_2_] induced the highest level of DNA damage at the first five telomeric repeats, T2-T6, compared to the other tested compounds. In comparison, less damage was observed at the two G3 sites for all compounds with damage not exceeding 3%, whereas the G5 site yielded a similar damage to the T6 and T7 sites for most compounds. Cisplatin induced the highest levels of DNA damage at the G4 and G5 sites, both accounting for over 8% of the total damage. Interestingly 4FH induced the highest proportion of DNA damage (approximately 6%) at the G4 site.

**Fig. 5:**
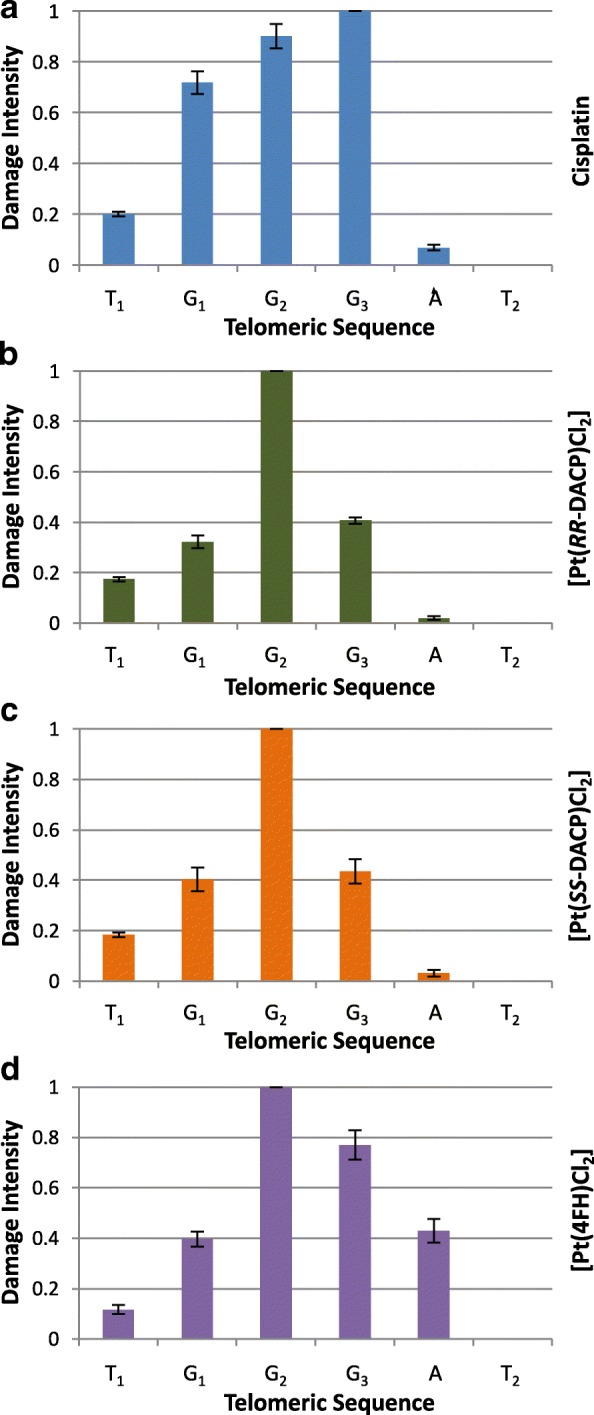
Bar Charts showing average damage at individual bases of the telomeric repeats. The graphs for (**a**) cisplatin, (**b**) [Pt(*RR*-DACP)Cl_2_], (**c**) [Pt(*SS*-DACP)Cl_2_] and (**d**) [Pt(4FH)Cl_2_], are highlighted in blue, green, orange and purple, respectively. The damage intensity of each individual peak was normalised to a maximal value of 1. The error bars represent the SEM determined across each of three replicates at the T4, T5 and T6 TGGGAT sequences

A subsequent analysis was conducted to further characterise the sequence specificity of adduct formation at the telomeric repeat sites. The damage intensities of each compound was averaged across the nucleotides in the telomeric repeat sites, T4, T5 and T6, since these produced the most consistent damage intensity across three repeat experiments. Cisplatin was found to induce the highest damage intensity at the third guanine (G_3_), followed by (in decreasing order) the second guanine (G_2_), first guanine (G_1_), first thymine (T_1_) and adenine (A), as shown in Fig. 5a. It can be noted that no damage was detected for cisplatin at the second thymine (T_2_). The novel compounds, [Pt(4FH)Cl_2_], [Pt(*RR*-DACP)Cl_2_] and [Pt(*SS*-DACP)Cl_2_], were found to exhibit a unique damage intensity profile compared to cisplatin, whereby G_2_ yielded the highest intensity of DNA damage rather than G_3_, as shown in Fig. 5b-d. It can be noted that [Pt(*RR*-DACP)Cl_2_] and [Pt(*SS*-DACP)Cl_2_] have almost identical profiles, with G_2_ yielding the highest damage intensity, followed by G_3_, G_1_, T_1_ and A, as depicted in Fig. 5b, c. Interestingly, the DNA damage profile of [Pt(4FH)Cl_2_] was the most distinct to that of cisplatin whereby the highest intensity was induced at G_2_, followed by G_3_, A, G_1_ and T_1_, as shown in Fig. 5d. Furthermore, only [Pt(4FH)Cl_2_] gave a damage intensity greater than 0.4 at the adenine (A) within the telomeric sequence.

### Interstrand cross-linking efficiency of the platinum-based compounds

**Fig. 6:**
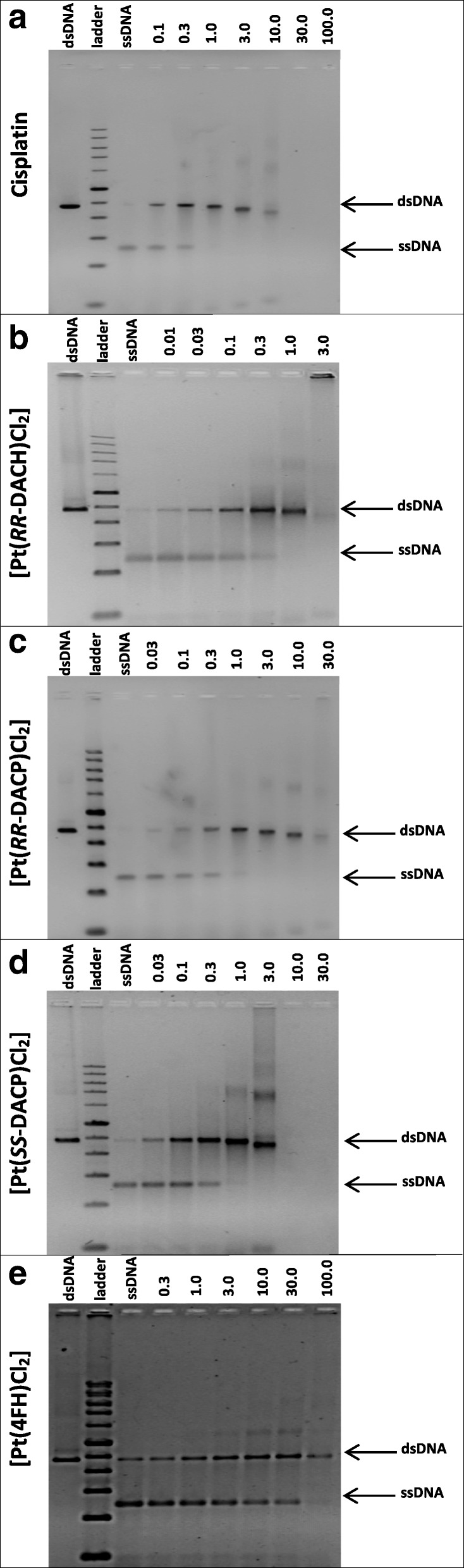
Images of 1.2% (v/v) denaturing agarose gels showing ssDNA or compound-induced cross-linked dsDNA. Denaturing agarose gels showing the large (2364 bp) *Pvu*II*-*cleaved fragment of pUC19 following treatment with (**a**) cisplatin, (**b**) [Pt(*RR*-DACH)Cl_2_], (**c**) [Pt(*RR*-DACP)Cl_2_], (**d**) [Pt(*SS*-DACP)Cl_2_], and (**e**) [Pt(4FH)Cl_2_]. DMF control samples of non-heat denatured (dsDNA) and heat denatured (ssDNA) plasmid are present on the left and right-hand side of the 1 kb ladder, respectively. Bands to the right correspond to heat-denatured samples that were prior treated with increasing concentration of compound (values are indicated in μM above each gel image)

The ability of each compound to induce DNA cross-linking was determined by measuring the extent to which treated dsDNA could be separated into ssDNA in the denaturing agarose gel assay, since cross-linking inhibits the separation of the dsDNA into ssDNA. DNA samples treated with only 5% (*v*/v) DMF solvent were included as controls and as expected exhibited complete separation into ssDNA in the denaturation assay. The interstrand cross-linking efficiency (ICLE) of each compound is shown in Table 1, whereby a higher ICLE value indicates a greater cross-linking efficiency. All tested compounds were found to induce DNA interstrand cross-linking to some extent, as seen in Fig. 6. Cisplatin treatment of approximately 1 μM completely inhibited the separation of the dsDNA, which correlated with an absence of the ssDNA form, shown in Fig. 6a. An analysis of the denaturing agarose gel revealed that 50% of the dsDNA form was retained at a cisplatin concentration of 0.09 ± 0.01 μM, correlating to a calculated ICLE value of 6.8 ± 0.5%, as shown in Table 1.

**Table 1:** Summary of compound-induced cross-linking of linear pUC19 DNA

Compound^a^	ICLE (%)	Drug concentration at 50% dsDNA retention (μM)	r_b_ (drug: nucleotide) at 50% dsDNA retention	Last detected data point (μM)
Cisplatin	6.8 ± 0.5	0.09 ± 0.01	1.9 × 10^− 3^ ± 1.4 × 10^− 4^	10
[Pt(*RR*-DACH)Cl_2_]	5.6 ± 2.1	0.13 ± 0.05	2.7 × 10^− 3^ ± 9.9 × 10^− 4^	3
[Pt(*RR*-DACP)Cl_2_]	3.1 ± 0.3	0.21 ± 0.02	4.1 × 10^− 3^ ± 3.9 × 10^− 4^	30
[Pt(*SS*-DACP)Cl_2_]	5.1 ± 0.2	0.12 ± 0.01	2.5 × 10^− 3^ ± 1.1 × 10^− 4^	3
[Pt(4FH)Cl_2_]	0.04 ± 0.02	23.52 ± 8.94	4.7 × 10^− 1^ ± 1.8 × 10^− 1^	300

^a^The r_b_ value was calculated based on 2 × 10^− 9^ mol of nucleotide sample incubated with each compound. The ‘last detected data point’ refers to the highest drug concentration at which the DNA was still detectable on the agarose gel. All values and their respective SEM values were determined from three separate experiment

The compounds, [Pt(*RR*-DACH)Cl_2_], [Pt(*RR*-DACP)Cl_2_] and [Pt(*SS*-DACP)Cl_2_], exhibited similar DNA cross-linking activity to that of cisplatin. Like cisplatin, [Pt(*RR*-DACH)Cl_2_] caused a complete absence of the ssDNA form at a drug concentration of 1 μM, see Fig. 6b. *RR*-DACH was found to induce a 50% retention of the dsDNA form at a drug concentration of 0.13 ± 0.05 μM, yielding an ICLE value of 5.6 ± 2.1%, as shown in Table 1. [Pt(*RR*-DACP)Cl_2_] and [Pt(*SS*-DACP)Cl_2_], were both found to induce a complete reduction in band intensity of the ssDNA form at a drug concentration of 3 μM, as seen in Fig. 6c, d, respectively. A 50% retention of the dsDNA form was attained by [Pt(*RR*-DACP)Cl_2_] and [Pt(*SS*-DACP)Cl_2_] at drug concentrations of 0.21 ± 0.02 μM and 0.12 ± 0.01 μM, respectively; correlating to ICLE values of 3.1 ± 0.3% and 5.1 ± 0.2%. The weakest interstrand cross-linking activity was exhibited by [Pt(4FH)Cl_2_]; inducing a 50% retention of the dsDNA form at a drug concentration of 23.52 ± 8.94 μM and yielding an ICLE value of 0.04 ± 0.02%. Furthermore, a complete reduction of the ssDNA form was not attained by [Pt(4FH)Cl_2_] until a drug concentration of over 100 μM was implemented; over 10-fold higher than that observed for the other tested compounds, as shown in Fig. 6e. Additionally there was DNA trapped in the loading wells of the denaturing agarose gels that was probably caused by extensive DNA cross-linking that resulted high molecular weight DNA or highly adducted DNA that was difficult to re-dissolve. This was particularly evident for [Pt(*RR*-DACH)Cl_2_], where a visible band at the top of the denaturing gel (at a drug concentration of 3 μM) can be seen in Fig. 6b. Further high molecular weight DNA cross-linking can be observed for [Pt(*SS*-DACP)Cl_2_] whereby a smearing and an additional band can be seen above the dsDNA form in Fig. 6d at a drug concentration of 3 μM. Further densitometry of these high molecular weight forms was not carried out and they were not seen in lanes where the 50% retention of the dsDNA form was noted.

### DNA unwinding induced by the platinum-based compounds

**Fig. 7:**
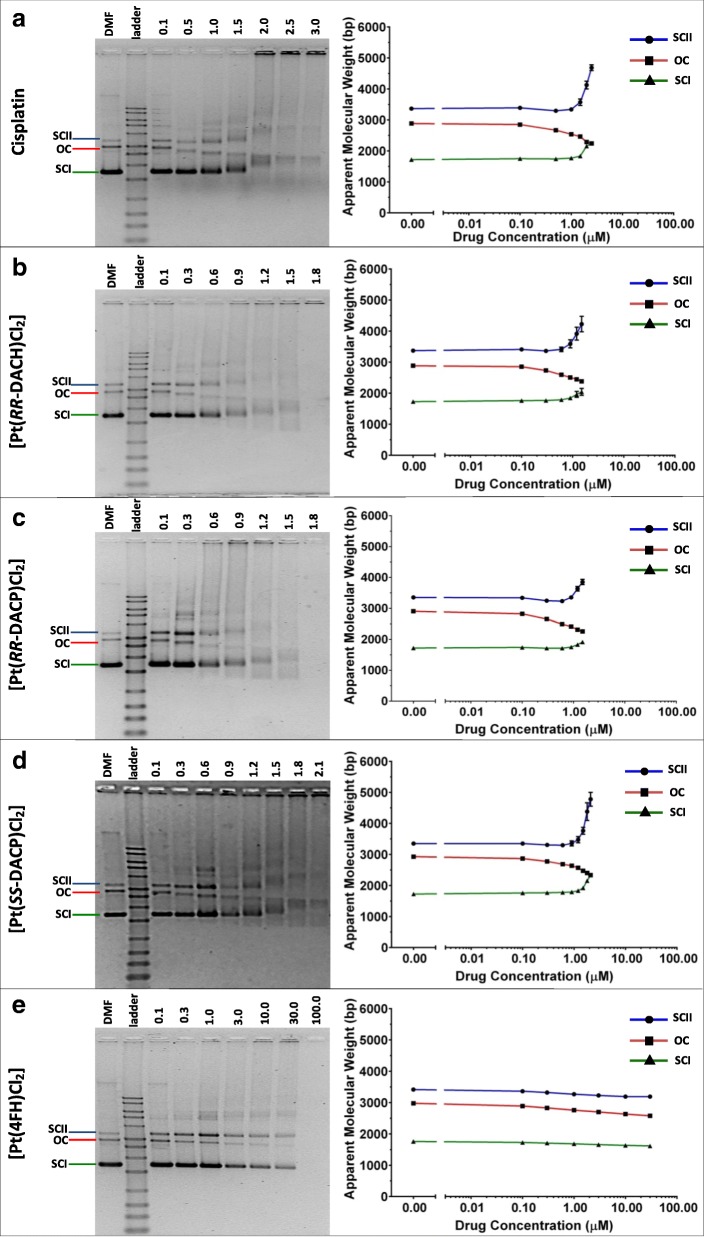
Images of 1% (v/v) native agarose gels and their respective semi-log scatter plots, showing changes in the apparent molecular weight of the native pUC19 DNA conformations following treatment with (**a**) cisplatin, (**b**) [Pt(*RR*-DACH)Cl_2_], (**c**) [Pt(*RR*-DACP)Cl_2_], (**d**) [Pt(*SS*-DACP)Cl_2_] and (**e**) [Pt(4FH)Cl_2_]. Bands corresponding to the OC, SCI and SCII conformations of pUC19, are indicated on the left-hand side of each gel image. The DMF solvent control sample (pUC19 without drug treatment) is situated to the left of the 1 kb ladder. All other lanes contain DNA samples treated with increasing concentration of compound as indicated in μM above each gel image. The changes in apparent molecular weight of each pUC19 conformation is plotted on the semi-log scatter graphs to the right of each image. The band migration for the OC, SCI and SCII plasmid conformations are represented by red, green and blue lines, respectively. The error bars shown represent the SEM determined across three separate experiments

The ability of each compound to unwind the supercoiled conformation of pUC19 was determined by electrophoretic mobility on native agarose gels. As expected, three different pUC19 conformations were observed on the agarose gels. These were the relaxed open circular form (OC) and the supercoiled forms I and II (SC I and SC II). It is thought that SC II is probably a dimer of SC I since its apparent molecular weight is approximately double that of the SC I form. These conformations resolve on agarose gels as distinct bands, as shown in Fig. 7.

Plasmid treatment with increasing concentrations of Cisplatin, [Pt(*RR*-DACH)Cl_2_], [Pt(*RR*-DACP)Cl_2_] or [Pt(*SS*-DACP)Cl_2_] up to 0.1 to 3 μM was found to induce a proportional decrease in electrophoretic mobility for the SC forms and an increase in that of the OC form, as shown in Fig. 7a-d. In contrast, [Pt(4FH)Cl_2_] caused minimal change to the electrophoretic mobility of the plasmid conformations across a broad concentration gradient ranging from 0.1 to 100 μM, as shown in Fig. 7e. Cisplatin and [Pt(*SS*-DACP)Cl_2_] caused the OC and SC I conformations to merge at a point of coalescence at drug concentrations of 2.5 and 2.4 μM, respectively. A similar trend was observed for [Pt(*RR*-DACH)Cl_2_] and [Pt(*RR*-DACP)Cl_2_] treated plasmid, although band intensity at the point of coalescence was faint within the treatment range, as shown in Fig. 7b, c. Again, this is because higher concentrations of compound treatment caused a build-up of DNA in the wells and reduced sample mobility. Again this may be due to extensive cross-linking or reduced solubility as mentioned earlier.

For the analyses of these data, the molar ratio of drug to nucleotide was calculated for the point of coalescence, this is referred to as r_b_(c). These r_b_(c) values were used to calculate the DNA unwinding angle induced by each compound. Cisplatin is known to induce an unwinding angle (ϕ) of 13° in plasmid DNA [36, 38], this value along with the experimentally determined r_b_(c) value was used to calculate that the SC I plasmid had a super-helical density value (σ) of − 0.073, using the formula described in the methods. This super-helical density value was used along with each r_b_(c) value to calculate the DNA unwinding angle induced by each compound, listed in Table 2. [Pt(*RR*-DACP)Cl_2_] induced the largest DNA unwinding angle (since its migration in the gel was reduced to a greater extent) followed by [Pt(*RR*-DACH)Cl_2_] and [Pt(*SS*-DACP)Cl_2_]. Minimal unwinding of the super-helical structure of the SC I plasmid was caused by [Pt(4FH)Cl_2_] under the experimental conditions. Although the point of coalescence was not observed on the gel, it was possible to calculate a small change in the DNA unwinding angle for [Pt(4FH)Cl_2_] by extrapolation of the data. However this value is reflective of an increase in migration of the OC band since little decrease in the migration of the SC I band was observed.

**Table 2:** Summary of compound-induced unwinding of the SC I conformation of pUC19

Compound	DNA unwinding angle (°)	Drug concentration at coalescence point (μM)^α^	r_b_(c) (drug: nucleotide at coalescence point)
Cisplatin	13	2.5 ± 0.0	1.0 × 10^− 1^ ± 2.0 × 10^− 3^
[Pt(*RR*-DACH)Cl_2_]	15.5 ± 1.9	2.2 ± 0.3	8.7 × 10^− 2^ ± 1.1 × 10^− 2^
[Pt(*RR*-DACP)Cl_2_]	16.9 ± 0.0	1.9 ± 0.0	7.8 × 10^− 2^ ± 2.0 × 10^− 4^
[Pt(*SS*-DACP)Cl_2_]	13.8 ± 0.6	2.4 ± 0.1	9.5 × 10^− 2^ ± 4.0 × 10^− 3^
[Pt(4FH)Cl_2_]	0.2 ± 0.0	184.5 ± 34.2	7.4 ± 1.4

^α^The drug concentration at the coalescence point indicates when the OC plasmid migrates at the same position as the SC I form. The r_b_ (c) values represent the drug to nucleotide ratio at the coalescence point. For all experiments, the amount of nucleotide sample incubated with each compound was 1 × 10^− 9^ mol. All values in the table were averaged from three separate experiments and are shown with their respective SEM values

### Cytotoxic activity of the platinum-based compounds in HeLa cells

The cytotoxicity was assessed in the cervical cancer-derived HeLa cells which are commonly used as an indicator of cellular activity. Cisplatin was utilised as a reference compound, with an IC_50_ value of 9.3 ± 0.2 μM being obtained in this investigation, which was similar to that previously determined for cisplatin in HeLa cells [39]. [Pt(*RR*-DACP)Cl_2_] was found to exhibit the highest degree of cytotoxic activity, having an IC_50_ value of 6.9 ± 1.0. The compounds, [Pt(*RR*-DACH)Cl_2_] and [Pt(*SS*-DACP)Cl_2_] were found to be less cytotoxic with IC_50_ values approximately twice that of [Pt(*RR*-DACP)Cl_2_], as shown in Table 3. [Pt(4FH)Cl_2_] was found to exhibit the weakest cytotoxic activity with an IC_50_ value of 28.8 ± 1.6 μM, which is approximately 4-fold higher than for [Pt(*RR*-DACP)Cl_2_]. Both the *SS* and *RR* isomer of [Pt(DACP)Cl_2_] were found to induce a complete loss in cell viability at a drug concentration of 50 μM whereas [Pt(*RR*-DACH)Cl_2_] required 100 μM. [Pt(4FH)Cl_2_] did not induce a complete loss in cell viability until a drug concentration of over 200 μM was applied. Cisplatin induced a complete loss in cell viability at a concentration of 30 μM which was the most effective of these compounds, as shown in Table 3.

**Table 3:** Summary of compound cytotoxicity in HeLa cells

Compound	IC_50_ (μM)^α^	Drug concentration at which complete cell death was obtained (μM)
Cisplatin	9.3 ± 0.2	30
[Pt(*RR*-DACH)Cl_2_]	12.3 ± 4.0	100
[Pt(*RR*-DACP)Cl_2_]	6.9 ± 1.0	50
[Pt(*SS*-DACP)Cl_2_]	13.7 ± 2.0	50
[Pt(4FH)Cl_2_]	28.8 ± 1.6	> 200

^α^These IC_50_ values represent the drug concentration at which a cell viability of 50% was obtained and were determined by non-linear regression. Each IC_50_ value was determined from the average of three separate experimental values and is reported along with its corresponding SEM value

## Discussion

### Sequence specificity of the platinum-based compounds in the pUC19/T7 sequence

All novel platinum-based compounds tested in this study similarly demonstrated a preference to covalently bind to guanine bases within the T7/*PvuII* sequence, particularly at the telomeric repeats (T2 to T7), G4, G3I and G3II and G5 sites. This binding pattern is in agreement with a previous investigation which reported that telomeric repeats (as well as other G-rich sequences) are effective binding targets for cisplatin [13]. This infers the presence of abundant 1,2 intrastrand GG DNA adducts, which is consistent with literature [4]. However, it was found that these compounds exhibit binding affinity with a preference for the G_2_ base of the telomeric repeats (as shown in Fig. 5), which was not observed for cisplatin. Both DACP isomers produced comparable DNA damage intensities to cisplatin, whereby a drug concentration of 0.3 μM was optimal to attain even DNA damage profiles distributed across the entire sequence. This could be because these compounds contain chloride groups in the *cis*-configuration and this trend has previously been observed for a *cis*-configured [Pt(1,2-diaminoethane)(Cl_2_)] [40]. Furthermore, [Pt(*RR*-DACP)Cl_2_] and [Pt(*SS*-DACP)Cl_2_] exhibited almost identical sequence specificity profiles at the telomeric repeats, shown in Fig. 5b, c. This was not surprising, since these compounds differ only in the conformation of the cyclopentane rings. A similar trend has been noted regarding the sequence specificity for the *RR* and *SS*-conformations of DACH [41]. The damage profiles of the two DACP isomers (and to a lesser extent cisplatin) exhibit a preference to bind the first three telomeric repeats in the T7/*PvuII* sequence, resulting in higher DNA damage intensities detected at the T2, T3 and T4 sites. It was intuitively thought that this bias towards the first cluster of telomeric repeats was due to excessive adduct formation inhibiting the *Taq* polymerase, which has previously been demonstrated for successively higher doses of cisplatin [12, 42]. However, it has been postulated that this may be because the 3′ end of the telomeric DNA repeats have an unusual conformation that exhibit an enhanced nucleophilicity [13]. This could explain the higher damage intensities detected at the T2, T3 and T4 sites in this current study, since they are situated proximally to the 3′ end of the T7/*PvuII* sequence in comparison to the other telomeric repeat sites.

Within the telomeric repeats, [Pt(4FH)Cl_2_] displayed a higher damage intensity at the A base that suggests a bias towards forming 1,2 GA intrastrand adducts. Additionally [Pt(4FH)Cl_2_] exhibited the weakest ability to form adducts and approximately 100-fold more, with respect to cisplatin, was required to obtain a detectable level of damage in the T7/*PvuII* sequence. The limited covalent binding ability of [Pt(4FH)Cl_2_] to DNA maybe due to steric hindrance of the bulky fluorobenzoic hydrazide-based ligands.

Drug resistance during cancer treatment has been correlated to cisplatin-based DNA adducts being commonly recognised and processed by DNA repair pathways [43]. Therefore, adducts of [Pt(*RR*-DACP)Cl_2_], [Pt(*SS*-DACP)Cl_2_] and[Pt(4FH)Cl_2_] may evade detection and overcome drug resistance. For instance, a human endometrial cancer cell line deficient in the repair protein hMSH2 exhibited less resistance to oxaliplatin than cisplatin and components of the mismatch repair protein complex were shown to bind with increased affinity to cisplatin-DNA adducts compared to oxaliplatin-adducts [44]. Additionally, it has been shown that MutS is able to recognise DNA adducts induced by cisplatin but not those of oxaliplatin [45].

### Interstrand cross-linking efficiency of the platinum-based compounds

In this study the ICLE value of cisplatin was 6.8% ± 0.5% which is in agreement with that previously determined [46]. [Pt(*RR*-DACP)Cl_2_], [Pt(*SS*-DACP)Cl_2_] and [Pt(*RR*-DACH)Cl_2_] were found to induce interstrand cross-links less efficiently and exhibited values of 3.1, 5.1 and 5.6%, respectively. [Pt(*RR*-DACH)Cl_2_] was most similar to cisplatin, consistent with values of 6% reported in a previous study [47]. The [Pt(*RR*-DACP)Cl_2_] isomer exhibited lower ICLE values than cisplatin and the [Pt(SS-DACP)Cl_2_] isomer in contrast to a previous investigation where the *RR*-isomer was marginally more active than the *SS*-isomer, with reported ICLE values of 5.7% ± 0.4 and 5.5% ± 0.5%, respectively [48]. Furthermore [Pt(*RR*-DACP)Cl_2_] was found to be more cytotoxic than [Pt(*SS*-DACP)Cl_2_] in HeLa cells suggesting that a high ICLE alone does not necessarily correlate to a high cytotoxicity.

The compound [Pt(4FH)Cl_2_] exhibited a poor interstrand cross-linking efficiency which is consistent with the previously described sequence specificity data whereby its DNA adducts were not detectable at low drug concentrations. This is possibly because it contains bulkier ligand groups that prevents the aquated ligands from binding to residues in the DNA helix. Steric hindrance of platinum drugs containing bulky ligand is a common drawback associated with their clinical development. Previously, the kinetic activity of sterically-hindered platinum(II) complexes were found to impede their nucleophilic substitution-based reactivity [49]. It is possible that similarly the bulky ligands of [Pt(4FH)Cl_2_] inhibit the nucleophilic substitution reaction at the N7 of the purine imidazole ring.

### DNA unwinding induced by the platinum-based compounds

Cisplatin, [Pt(*RR*-DACH)Cl_2_], [Pt(*RR*-DACP)Cl_2_] and [Pt(*SS*-DACP)Cl_2_] were each able to induce unwinding of the SC I conformation of the pUC19 plasmid decreasing its electrophoretic mobility. Furthermore, these compounds were found to cause an increase in the electrophoretic mobility of the OC plasmid conformation. This trend has been previous reported [50] and it is thought that such bifunctional adducts cause the native OC DNA helix to condense [51] and migrate faster through the agarose gel.

The point of coalescence of the SC I and OC plasmids conformations were detected for cisplatin at an r_b_(c) value of approximately 0.1. This value is marginally higher than those previously reported of 0.076 [36] and 0.08 [52]. The higher r_b_(c) determined for cisplatin in this study can be attributed to the high super helical density of the template. For instance, pUC19 template in this study had a super helical density of − 0.073 which is larger than the − 0.055 value previously reported [36]. A higher density would require a higher concentration of cisplatin to induce unwinding.

The compounds [Pt(*RR*-DACH)Cl_2_], [Pt(*RR*-DACP)Cl_2_] and [Pt(*SS*-DACP)Cl_2_] exhibited DNA unwinding angles of 16.9° and 15.5° and 13.8°, respectively. These were larger than the 13° unwinding angle known for cisplatin. The unwinding angle determined for [Pt(*RR*-DACH)Cl_2_] in this study is in agreement with that of 15° previously reported [47]. Additionally, it was suggested that the unwinding ability of [Pt(*RR*-DACH)Cl_2_] may impede the removal of these bulky adducts by the DNA repair system. [Pt(*RR*-DACP)Cl_2_] was found to be more effective than the *SS*-isomer by a factor of approximately 1.2. This trend has also been observed for the *RR*-DACH isomer over its *SS*-counterpart [53]. Molecular modelling analysis has shown that the two ammonia ligands of the [Pt(*SS*-DACH)Cl_2_] complex are more prone to clashes with the DNA structure than the [Pt(*RR*-DACH)Cl_2_] complex [53]. A similar mechanism may explain the differing DNA unwinding efficiencies of the DACP isomers reported here.

Weak DNA unwinding was exhibited by [Pt(4FH)Cl_2_] which caused only minimal changes to the electrophoretic mobility of pUC19. The chemical structure of [Pt(4FH)Cl_2_] consists of two bifunctional chloride groups so that it could covalently bind to DNA to induce unwinding. However, the reduced unwinding ability of [Pt(4FH)Cl_2_] may be attributed to its bulky fluorobenzoic hydrazide-based ligand, which physically hinders its ability interact with DNA. This is in keeping with reduced DNA unwinding as a consequence of steric hindrance for compounds containing bulky naphthalene moieties around the platinum group [54].

### Cytotoxic activity of the platinum-based compounds in HeLa cells

In this investigation the IC_50_ value of cisplatin was determined to be 9.3 ± 0.2 μM which is consistent with that of 11.42 μM determined for cisplatin in HeLa cells [39]. Generally, [Pt(*RR*-DACP)Cl_2_], [Pt(*RR*-DACH)Cl_2_] and [Pt(*SS*-DACP)Cl_2_] induced IC_50_ values within the same order of magnitude as cisplatin, at 6.9, 12.3 and 13.7 μM, respectively. This is likely attributed to the fact that they are all *cis-*configured covalent binders containing cyclohexane or cyclopentane ligands. [Pt(*RR*-DACH)Cl_2_] was slightly less cytotoxic to HeLa cells than cisplatin which is in agreement with a previous study, whereby DACH-derived compounds were tested in four different cancer cell lines [55]. The complex of the *RR*-isomer of DACP was approximately 2-fold more active than the *SS*-isomer. Similarly it has been shown that the *RR*-isomer of oxaliplatin is more active than its SS-isomer [56]. This is not surprising considering that both DACP isomers contain a comparable cycloalkane ligand to oxaliplatin, albeit that the DACP cyclic ligand contains five carbons whereas the DACH ligand of oxaliplatin contains six. Furthermore, it has been reported that the cytotoxic activity of platinum-based complexes containing *RR*-DACP-based ligands in mouse leukemia cells exhibited greater cytotoxicity activity than the *SS*-isomers [57]. However, this trend does not hold true for all *RR*-configured platinum-based compounds; for instance [(1,10-phenthronline)(1*R*,2*R*-diaminocyclohexane) platinum(II) dichloride] (PHENRR), [(5-methyl-1,10-phenthronline)(1*R*,2*R*-diaminocyclohexane) platinum(II) dichloride] (5MERR) and [(3,4,7,8-tetramethyl-1,10-phenthronline)(1*R*,2*R*-diaminocyclohexane) platinum(II) dichloride] (3478MERR) are less active than their respective SS-configured isomers, PHENSS, 5MESS and 3478MESS in human colon and ovarian cell lines [56]. Clearly, it is not possible to predict with certainty the cytotoxic effects of novel platinum-based compounds and each needs to be tested empirically. Such an approach is crucial for the future design and development of novel chemotherapeutic compounds.

## Conclusion

DNA sequence specificity analysis revealed that DNA adducts at telomeric repeat sequences and other G-rich sequences are effective targets for cisplatin and other novel compounds. The isomeric compounds, [Pt(*RR*-DACP)Cl_2_] and [Pt(*SS*-DACP)Cl_2_], induced a relatively similar amount of DNA damage comparable to that of cisplatin, whereas, [Pt(4FH)Cl_2_] was found to be considerably less efficient. We have proposed that the lesser activity of [Pt(4FH)Cl_2_] is likely due to steric hindrance associated with its bulky fluorobenzoic hydrazide ligands. Interestingly, [Pt(*RR*-DACP)Cl_2_], [Pt(*SS*-DACP)Cl_2_], and [Pt(4FH)Cl_2_] exhibited a slight variation in sequence specificity at the telomeric repeat sequences compared to cisplatin. Despite exhibiting marginally lower interstrand cross-linking efficiencies than cisplatin, all of the tested compounds (except for 4FH) were able to inducing a greater degree of structural distortion, as indicated by the DNA unwinding efficiency. Additionally, [Pt(*RR*-DACP)Cl_2_] exhibited a relatively higher cytotoxic activity than cisplatin in HeLa cells. Overall, these data provide an insight into the activity of these compounds towards DNA and in relation to assessing their antitumour properties. Further assessment of these compounds in a range of other cell lines, in relation to their cellular DNA binding and cytotoxic activity, is warranted to further predict their clinical potential.

## Abbreviations

56MESS: [(5,6-dimethyl-1,10-phenanthroline) (1S,2S-diaminocyclohexane)platinum(II)]^2+^
[Pt(4FH)Cl_2_]: [(4-fluorobenzoic hydrazide)-platinum(II) dichloride]
[Pt(*RR*-DACH)Cl_2_]: [(1*R*,2*R*-diaminocyclohexane)-platinum(II) dichloride]
[Pt(*RR*-DACP)Cl_2_]: [(1R,2R-diaminocyclopentane)-platinum(II) dichloride]
[Pt(*SS*-DACP)Cl_2_]: Dichloro(1S,2S-diaminocyclopentane)-platinum(II)
Cisplatin: *cis*-Diamminedichloroplatinum(II)
DMF: Dimethylformamide
ICLE: Interstrand cross-linking efficiency
LAR: Linear amplification reaction
OC: Open circular
r_b_: Drug to nucleotide ratio
r_b_(c): Drug to nucleotide ratio at the point of coalescence
SCI: Supercoiled conformation I
SCII: Supercoiled conformation II
